# Evaluating the Utility of ctDNA in Detecting Residual Cancer and Predicting Recurrence in Patients with Serous Ovarian Cancer

**DOI:** 10.3390/ijms241814388

**Published:** 2023-09-21

**Authors:** Jie Wei Zhu, Fabian Wong, Agata Szymiczek, Gabrielle E. V. Ene, Shiyu Zhang, Taymaa May, Steven A. Narod, Joanne Kotsopoulos, Mohammad R. Akbari

**Affiliations:** 1 Women’s College Research Institute, Women’s College Hospital, University of Toronto, Toronto, ON M5S 1B2, Canada; 2 Department of Medicine, McMaster University, Hamilton, ON L8P 1H6, Canada; 3 Institute of Medical Science, Faculty of Medicine, University of Toronto, Toronto, ON M5S 1A8, Canada; 4 Division of Gynecologic Oncology, Department of Surgical Oncology, Princess Margaret Cancer Center, University Health Network, Toronto, ON M5G 2M9, Canada; 5 Division of Gynecologic Oncology, Department of Obstetric and Gynecology, University of Toronto, Toronto, ON M5G 2C4, Canada; 6 Dalla Lana School of Public Health, University of Toronto, Toronto, ON M5T 3M7, Canada

**Keywords:** circulating tumour DNA, liquid biopsy, ovarian cancer, minimal residual disease, prognosis

## Abstract

Ovarian cancer has a high case fatality rate, but patients who have no visible residual disease after surgery have a relatively good prognosis. The presence of any cancer cells left in the peritoneal cavity after treatment may precipitate a cancer recurrence. In many cases, these cells are occult and are not visible to the surgeon. Analysis of circulating tumour DNA in the blood (ctDNA) may offer a sensitive method to predict the presence of occult (non-visible) residual disease after surgery and may help predict disease recurrence. We assessed 48 women diagnosed with serous ovarian cancer (47 high-grade and 1 low-grade) for visible residual disease and for ctDNA. Plasma, formalin-fixed paraffin-embedded (FFPE) tumour tissue and white blood cells were used to extract circulating free DNA (cfDNA), tumour DNA and germline DNA, respectively. We sequenced DNA samples for 59 breast and ovarian cancer driver genes. The plasma sample was collected after surgery and before initiating chemotherapy. We compared survival in women with no residual disease, with and without a positive plasma ctDNA test. We found tumour-specific variants (TSVs) in cancer cells from 47 patients, and these variants were sought in ctDNA in their post-surgery plasma. Fifteen (31.9%) of the 47 patients had visible residual disease; of these, all 15 had detectable ctDNA. Thirty-one patients (65.9%) had no visible residual disease; of these, 24 (77.4%) patients had detectable ctDNA. Of the patients with no visible residual disease, those patients with detectable ctDNA had higher mortality (20 of 27 died) than those without detectable ctDNA (3 of 7 died) (HR 2.32; 95% CI: 0.67–8.05), although this difference was not statistically significant (*p =* 0.18). ctDNA in post-surgical serum samples may predict the presence of microscopic residual disease and may be a predictor of recurrence among women with ovarian cancer. Larger studies are necessary to validate these findings.

## 1. Introduction

Ovarian cancer is the fifth leading cause of death from cancer in Canadian women [1]. High-grade serous ovarian cancer accounts for over 80% of all ovarian cancer deaths [2,3,4]. The standard treatment for high-grade serous ovarian cancer is a combination of surgery and chemotherapy [5,6,7]. Most patients have an initially favourable response to therapy; however over one-half of patients will experience cancer recurrence [6,7]. The estimated three-year survival following a recurrence is about 3% [8]. Therefore, prevention of ovarian cancer recurrence is directly associated with the prevention of death. 

The most important predictor of recurrence is the absence of any residual tumour following primary debulking surgery [2]. Patients are classified according to the presence or absence of “visible” residual disease [9]. However, this method is subjective and relies on visual inspection by the surgeon [9,10]. There may be residual (microscopic) cells that are not observed and thus, a proportion of patients will be inappropriately classified as having no residual disease when in fact, there are malignant cells present [9,10,11]. These patients may be at high risk of recurrence and death [9].

One way of identifying microscopic residual disease includes cytologic analysis of the peritoneal washings [12,13]. Given that peritoneal dissemination occurs frequently in ovarian tumours, microscopic intraperitoneal metastases can be detected with aspiration of free abdominal fluid during surgery and sending this sample for cytologic examination [12]. Findings from studies conducted among patients with localized ovarian cancer undergoing primary debulking surgery demonstrated that peritoneal washing cytology provides better prognostic information than does stage alone [12,13,14]. Despite this, peritoneal washings are not performed routinely for all ovarian cancer patients. 

A second method for detecting microscopic residual disease is a liquid biopsy. Liquid biopsy is a non-invasive technique that is based on analyzing circulating free DNA (cfDNA). Circulating tumour DNA (ctDNA) is released into the blood when tumour cells die and is detectable in the cfDNA extracted from plasma [15,16]. ctDNA fragments released by cancer cells carry the same structural sequence and epigenetic variations as do the tumour cells [16]. As a result, the presence of tumour-specific DNA genetic alterations in the serum post-surgery may be used as an indicator of the persistence of tumour cells and has the potential to be a cancer biomarker [17].

The primary objective of this study was to evaluate if the detection of ctDNA could be employed to accurately classify residual disease status after primary debulking surgery. We also sought to determine whether the presence of ctDNA after primary debulking surgery (and before chemotherapy) is correlated with cancer recurrence and patient survival.

## 2. Results

Among the 48 women included in this study, the mean age at diagnosis was 58.9 years. The majority were diagnosed with Stage III disease (85.4%). All but one patient had high-grade disease. Most patients (*n* = 45, 93.8%) did not receive neoadjuvant chemotherapy (i.e., before debulking surgery), while three patients (6.2%) had unknown treatment status. After surgery (and prior to chemotherapy), 32 (66.7%) of the patients were classified by surgical inspection as having no visible residual disease, 15 (31.3%) patients were classified as having visible residual disease, and one patient had unknown residual disease status (Table 1).

### 2.1. Germline Mutations

Among the 48 patients from whom germline sequencing data were obtained, 22 (45.8%) were carriers of a pathogenic germline mutation. The most frequently mutated genes were *BRCA1* (*n* = 12), followed by *BRCA2* (*n* = 7), *RAD51C* (*n* = 2), and *RAD50* (*n* = 1) (Table 2). Two of the patients with *BRCA1* and *RAD51C* mutations also had a pathogenic mutation in *CHEK2* and *MSH6* genes, respectively. Loss of heterozygosity (LOH) of the pathogenic germline variant was observed in the tumour DNA for 21 of the 22 patients (95.5%) (the single exception was *RAD50*).

### 2.2. Tumor Mutations

Using the panel of 59 genes sequenced in matched tumour and germline DNA, tumour-specific variants (TSVs) were found in 47 (97.9%) of the patients. Pathogenic *TP53* mutations were identified in the tumour DNA of 27 (56.3%) patients. We also identified pathogenic *BRCA2* mutations in six patients and *BRCA1* mutations in two patients. One patient had both a *BRCA1* and *BRCA2* somatic mutation, and three patients had germline *BRCA1* or *BRCA2* mutations as well (Table 3). One patient had pathogenic mutations in each of *BRAF*, *BRIP1*, *MSH2* and *RB1* (Table 4). There was one patient who had no TSVs and thus could not be analyzed for ctDNA.

### 2.3. ctDNA in Pre- and Post-Surgery Plasma Samples

**Figure 1:**
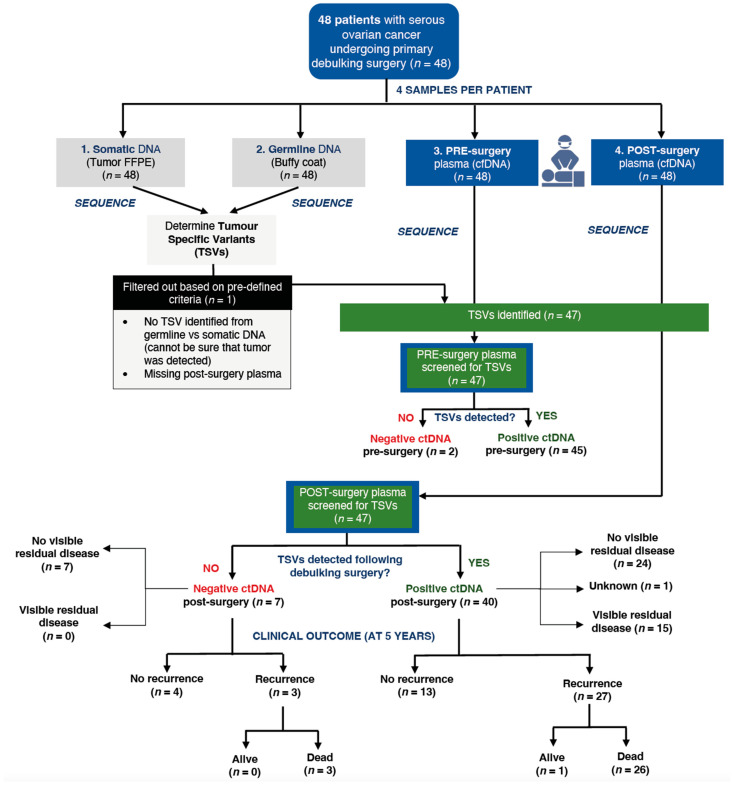
Flow of enrolled participants from collected samples to final clinical outcome.

Among the 47 post-surgery plasma samples, 40 samples (85.1%) had detectable ctDNA and seven samples (14.9%) did not have ctDNA. Of the seven patients who did not have detectable ctDNA, four patients (57.1%) had no disease recurrence and were alive in the last follow-up, while the other three patients had recurrent disease and subsequently died. All seven patients with no ctDNA were classified as having no visible residual disease post-surgery (Figure 1). 

For the 40 post-surgery samples with detectable ctDNA, 27 (67.5%) patients had recurrent disease, of whom 26 (65.0%) died; 13 (32.5%) patients had no recurrence and were alive in the last follow-up (Figure 1). 

Of the 15 patients with visible surgical residual disease, all had ctDNA detected. Patients with detectable ctDNA in post-surgery samples had a higher mortality risk compared to those without detectable ctDNA (HR = 2.27; 95% CI: 0.68–7.56), although this difference was not statistically significant (*p* = 0.17). Five-year survival was 85.7% for those with no detectable ctDNA, compared to 56.8% for those with detectable ctDNA (Figure S1).

**Figure 2:**
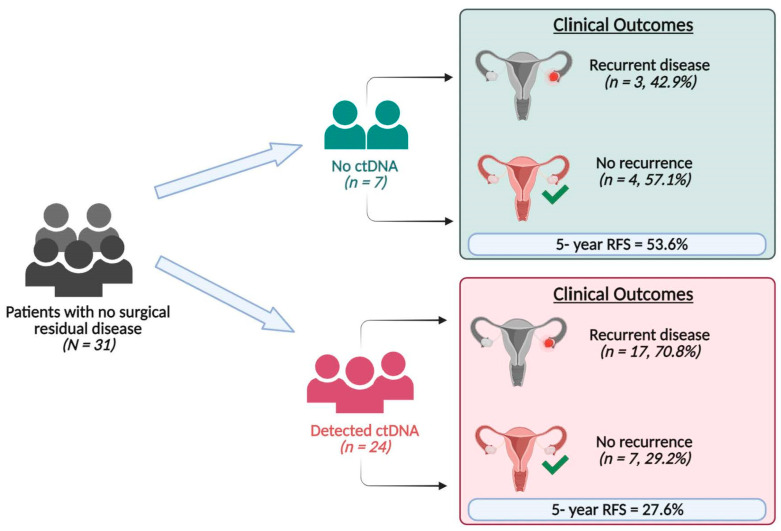
Results of ctDNA analysis of post-surgery plasma samples of patients with no surgical residual disease and their clinical outcome.

Of the 31 patients with no surgical residual disease, 24 (77.4%) had detectable ctDNA and seven (22.6%) had no detectable ctDNA (Figure 2). Of the 24 patients with detectable ctDNA, 17 (70.8%) had recurrence, and 16 died. Of the seven patients with no detectable ctDNA, three (42.9%) had recurrence, and all three died. Patients classified as having no surgical residual disease who had detectable post-surgery ctDNA had a higher mortality risk compared to those who did not have detectable post-surgery ctDNA (HR = 2.32; 95% CI: 0.67–8.05, *p =* 0.18), with a five-year survival rate of 85.7% for those with undetectable ctDNA vs. 58.3% for those with detectable ctDNA (Figure S2). 

The five-year recurrence-free survival for patients without surgical residual disease and with detectable post-surgical ctDNA was 27.6% compared to 53.6% in those without detectable ctDNA (HR = 2.06; 95% CI: 0.60–7.10, *p* = 0.23) (Figure S3).

## 3. Discussion

In this study of 48 women diagnosed with ovarian cancer, we identified tumour-specific variants (TSVs) by comparing germline mutations in WBCs and somatic mutations in FFPE samples in a panel of 59 ovarian cancer driver genes. For most patients, these mutations were detectable in presurgical serum specimens. We then sought to see if the same mutations were present in the blood after surgery was completed. We assumed that the presence of the mutations in the blood post-surgery was indicative of residual cancer cells left after surgery. We then conducted a survival analysis to see if cancer recurrence and survival were associated with the presence of ctDNA post-surgery. 

Most patients who were classified by the surgeon as having no visible residual disease following primary debulking surgery were, in fact, found to have detectable ctDNA in their post-surgery plasma. 

Among seven patients who did not have detectable ctDNA, three patients developed disease recurrence and died (Figure 2), compared to 16 of 24 patients with detectable post-operative ctDNA who developed disease recurrence and died. These data highlight the potential benefit of using ctDNA as a surrogate for detecting microscopic residual disease after surgery.

In a longitudinal study of 27 colorectal patients, all 14 patients with detectable ctDNA immediately after surgical resection eventually relapsed during the three-year follow-up period, compared to those without detectable ctDNA, all of whom remained cancer-free [18]. A prospective study with 23 ovarian cancer patients evaluating the ability of serially collected ctDNA to screen for disease recurrence following primary debulking surgery reported that the sensitivity of ctDNA in predicting tumour recurrence was 91% [17]. The authors also found that detectable ctDNA in the plasma predicted recurrence on average 7 months earlier than CT imaging [19]. Another exploratory retrospective study with 20 high-grade serous ovarian cancer patients used TP53 mutations detected by sequencing of cfDNA extracted from serum following primary debulking surgery and found that a greater proportion of patients with non-optimal debulking (67%) had detectable ctDNA (cfDNA with TP53 mutations) compared to patients with optimal debulking (45%) [20]. Although evaluation for recurrence and metastasis currently relies on CA-125 and CT, the use of ctDNA may offer more dynamic and timely monitoring of recurrence in ovarian cancer. 

Few studies have evaluated ctDNA as a prognostic biomarker in ovarian cancer. One prospective study examining serial plasma samples for 22 high-grade serous ovarian cancer patients reported that undetectable ctDNA levels at 6 months following primary debulking surgery and adjuvant treatment were associated with significant improvement in overall survival (*p* < 0.05) and progression-free survival (*p =* 0.001) [19]. Another prospective study of *TP53* mutations in cfDNA of 61 high-grade serous ovarian cancer patients demonstrated that patients with high levels of detectable ctDNA (≥0.2 copies/μL), 3 months after completing adjuvant chemotherapy had a significantly higher risk of recurrence of 58.3% compared to 6.7% in patients with low ctDNA levels (<0.2 copies/μL) [21]. To our knowledge, the present study is the first to evaluate how the detection of ctDNA after primary debulking surgery and prior to chemotherapy can predict microscopic residual disease among patients with no surgical residual disease and be a possible predictor of worse survival. However, the difference in survival in our study did not achieve statistical significance given the limited sample size. Previous studies mostly provided data on using ctDNA analysis for determining real-time tumour status post-operatively in advanced-stage ovarian cancer as a non-invasive method of monitoring disease recurrence. We found germline *BRCA1*/2 mutations in 39.6% of our patients. The reason for this is not clear but may reflect that the selection of patients for the biobank favors *BRCA1* and *BRCA2* carriers. 

We found pathogenic somatic *TP53* mutations in 27 (56.3%) of 48 participants. This is lower than *TP53* mutation rates of up to 96.7% in HGSOC reported by Ahmed et al. [22], but is consistent with recent, smaller studies reporting 50–80% of ovarian cancers harbor somatic *TP53* mutations [23,24]. Our findings of a lower frequency of somatic *TP53* mutations may be attributable to possible selection bias given the smaller sample size. Our assay covered 100% of *TP53* coding exons and 25 bp in their adjacent introns at 500x and higher depth of coverage. As such, no *TP53* mutation was expected to be missed in any tumour sample.

### 3.1. Study Limitations

Our sample size was small, and study power was limited. We included post-surgical samples collected at one timepoint following debulking surgery and prior to initiating adjuvant chemotherapy. Our results represent a cross-sectional view of tumour burden immediately following surgery, when recurrent microscopic disease is minimal and ctDNA is, therefore, more difficult to detect. Serial plasma samples collected throughout the first 2 years after surgery may help to more accurately assess the ability of ctDNA to monitor response to adjuvant chemotherapy over time. Future research may also examine whether there is a difference in recurrence rates and patient survival between different detectable TSVs. Larger, well-powered studies are warranted to further elucidate our findings.

Another inherent limitation of our study is the possible false positive results. Although ctDNA has high specificity in ovarian cancer [25,26], the previous literature has suggested false positive findings may occur due to clonal hematopoiesis of indeterminate potential (CHIP) [27]. CHIP is a benign clonal expansion of hematopoietic stem cells that is commonly associated with age and deep sequencing of broad gene panels [28]. The most frequently detected CHIP-related mutations reported are *DNMT3A*, *TET2*, *ASXL1*, *TP53* and *JAK2* [27,29]. To avoid overtreatment on the basis of a single positive result alone, clinicians should be aware that these mutations in plasma may occur even in healthy individuals and may increase false positive rates of ctDNA detection. However, CHIP will be less problematic when we know the TSVs of a patient. Therefore, although we cannot completely rule out the appearance of exact same mutations as the TSVs through CHIP, this will be very unlikely. That is why, in this study, we first sequenced matched tumour-germline DNA for determining TSVs for each individual and then searched those mutations in cfDNA rather than searching for mutations in these genes blindly. The studies examining the impact of CHIP on false positive rates of ctDNA detection were conducted for esophageal, colorectal, and non-small cell lung cancer, and limited data exist for ovarian cancer. To our knowledge, there is currently no standard metric to quantify the accuracy of ctDNA as an indicator for residual disease status, and future research is required to prevent the clinical overtreatment of false positive cases.

### 3.2. Implications and Contributions to Knowledge

The ability to detect tumour DNA in blood plasma has the potential to transform the management of ovarian cancer. As we move into a rapidly evolving era towards precision medicine, the reliance on visual inspection for residual tumour is suboptimal; liquid biopsies represent a minimally invasive and sensitive method to test for residual cancer cells. Future efforts should be directed towards developing standardized methods that are optimized for ctDNA detection from peripheral blood and both efficient and cost-effective. Our study requires replication under various scenarios. Nonetheless, the results of this study highlight the clinical value of using ctDNA in the care of patients with high-grade serous ovarian cancer. 

## 4. Methods and Materials

### 4.1. Sample Population

**Figure 3:**
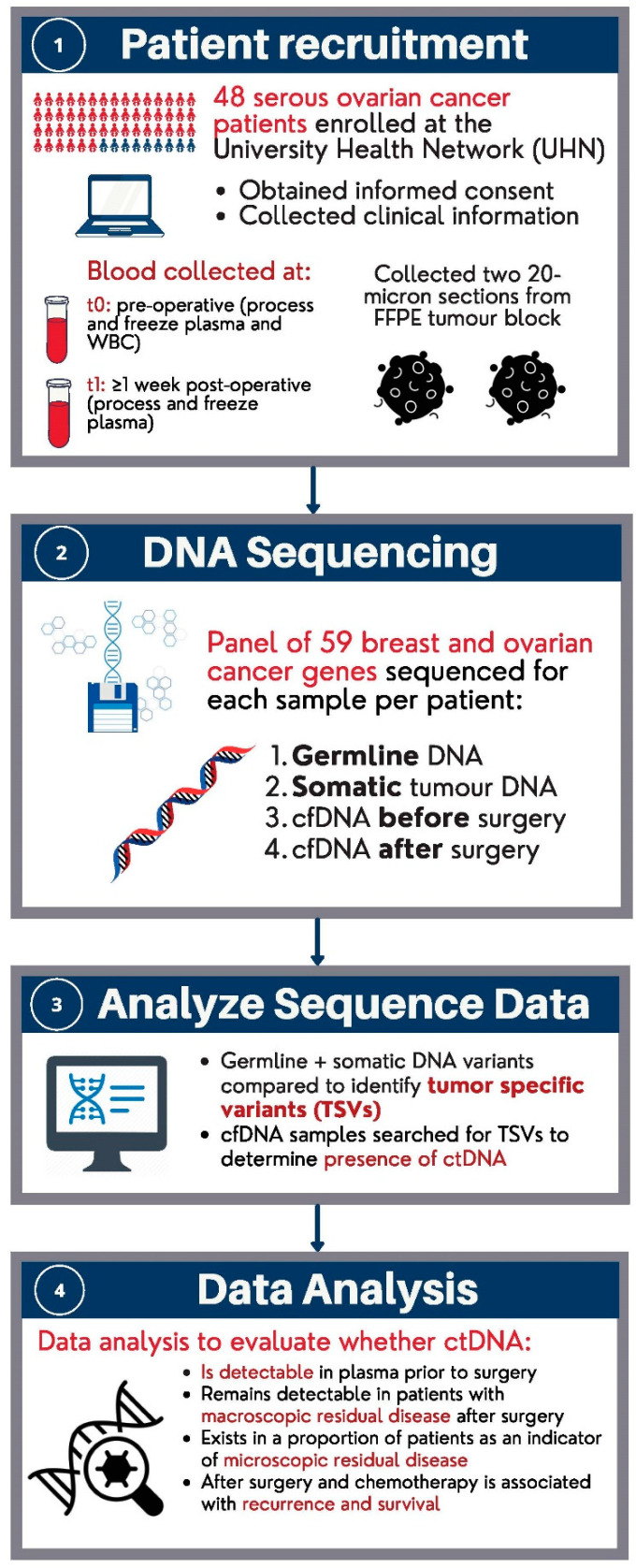
Overview of study design.

An overview of the study population, sample collection and their analysis are summarized in Figure 3. We included samples from patients diagnosed with stage I–IV high-grade serous ovarian cancer who received primary debulking surgery and chemotherapy at the University Health Network (UHN). Patients were diagnosed between 2011 and 2015. The study population consisted of 48 patients (*n* = 48) that were classified as either “having” or “not having” visible residual disease by visual inspection of the surgeon or “unknown”, after primary debulking surgery. All of the patients provided informed consent to use their samples for cancer research at the time of biobanking. Figure 3 below is an overview of the study design.

The collection of all biological specimens was carried out in accordance with the UHN Gynecology Biobank Protocol. Tumour tissue specimens were obtained during surgery and stored in formalin-fixed paraffin-embedded (FFPE) blocks until further use for isolation of the somatic tumour DNA. A blood sample was collected before the surgery. Ten millilitres of blood were collected from each patient in purple-top blood tubes. This blood sample was used for extracting DNA from white blood cells as the representative of germline DNA and for extracting cfDNA from plasma used for pre-surgery ctDNA analysis. We also collected a plasma sample 2 weeks after surgery and before starting chemotherapy for extracting cfDNA used for the post-surgery ctDNA analysis. All samples were analysed by sequencing within 5 years of patient diagnosis and blood collection.

### 4.2. Laboratory Methods

DNA from plasma (cfDNA), FFPE tumour tissue blocks (somatic tumour DNA), and white blood cells (germline DNA) were extracted using automated extraction and purification with the QIAsymphony instrument and QIAsymphony DSP DNA and circulating DNA kits. Tumour blocks with at least 40% tumour cellularity were chosen for DNA extraction. A pathologist determined the tumour area on the block section to macrodissect before DNA extraction. Four millilitres of plasma were used in the QIAsymphony cfDNA extraction, and the purified cfDNA was eluted in 25 μl of dilution buffer. All DNA samples were sequenced for a panel of 59 breast and ovarian cancer drivers and/or susceptibility genes based on the COSMIC Cancer Gene Census [30], using the SureSelect XT assay from Agilent for target enrichment and Illumina NextSeq for sequencing. The 59 genes included in our assay were *AKT1*, *AKT2*, *APOBEC3B*, *ARID1A*, *ARID1B*, *ATM*, *ATR*, *BARD1*, *BRAF*, *BRCA1*, *BRCA2*, *BRIP1*, *CASP8*, *CCND1*, *CDH1*, *CDKN1B*, *CHEK2*, *CTCF*, *CTNNB1*, *EP300*, *ERBB2*, *ESR1*, *FES*, *FOXA1*, *FOXL2*, *GATA3*, *KEAP1*, *LRP1B*, *MAP2K4*, *MAP3K1*, *MAP3K13*, *MAPK1*, *MLH1*, *MSH2*, *MSH6*, *NBN*, *NCOR1*, *NOTCH1*, *PALB2*, *PBRM1*, *PIK3CA*, *PIK3R1*, *PMS2*, *PPM1D*, *PPP2R1A*, *PTEN*, *PTPRT*, *RAD50*, *RAD51C*, *RAD51D*, *RB1*, *RECQL*, *RNF43*, *SALL4*, *SMARCD1*, *STK11*, *TBX3*, *TP53*, and *XRCC2*. Currently, germline and tumour DNA of ovarian cancer patients are tested for many of the genes included in our 59 gene assays to determine which patients may potentially benefit from PARP inhibitors [31,32,33]. We included some additional commonly mutated genes in ovarian tumours to increase the chance of detecting tumour-specific variants (TSVs). As such, the same assay used for analyzing germline and tumour DNA may be used for analyzing cfDNA and more likely be adopted in the clinical setting.

For the cfDNA and somatic tumour DNA, we used the high-sensitivity (HS) version of the SureSelect XT assay, which utilizes molecular barcodes that allowed us to detect mutations with a very low allele frequency (≤1%). Initial quantification and quality control of the prepared libraries were performed with high-sensitivity kits using both TapeStation and Qubit. Paired-end sequencing with sequence reads of 150 bp was performed for all of the sequences on the Illumina NextSeq sequencer. For sequencing of germline DNA, we targeted 200× depth of coverage. For the tumour cell DNA sequencing, we targeted 1000× depth of coverage with a starting amount of 200 ng of DNA template. This allowed for the detection of mutations in tumour sub-clones with cell fractions as low as 1%. For sequencing of the cfDNA, we started with at least 10 ng of cfDNA template and targeted 10,000× depth of coverage. This allowed us to detect tumour-specific mutations with variant allele frequencies of 1% or less among cfDNA. We have validated our gene panel and laboratory assay for detecting mutations in commercial control samples with known allele frequencies of 0.25% and 1% in several genes, including *P53*, *PTEN*, *ATM*, *EGFR*, *KRAS* and *PIK3CA*.

### 4.3. Data Analysis

#### DNA Sequencing and Bioinformatics Analysis

The sequencing data for the germline DNA and somatic tumour DNA for each patient were compared to identify tumour-specific variants (TSVs) among 59 breast and ovarian cancer-related genes. We searched for the TSVs in the cfDNA sequences to detect ctDNA. The detection of the TSVs in the cfDNA was an indicator for the existence of ctDNA in the plasma, which indicates the presence of ovary tumour cells in the body.

Sequence reads were aligned to the human genome’s reference sequence using Burrows–Wheeler Aligner incorporated into the Sentieon Package (Sentieon Inc. San Jose, CA, USA) [34]. The same package was used to convert the sequence alignment map (SAM) files to BAM format and to sort and index the BAM files. All of the unmapped reads, reads aligned to more than one human genome region, and duplicate reads were filtered out from the BAM file in the next step. AGeNT software (https://www.agilent.com/en/product/next-generation-sequencing/hybridization-based-next-generation-sequencing-ngs/ngs-software/agent-232879, accessed on 17 September 2023) from Agilent was used for dealing with molecular barcodes before variant calling [35]. The Haplotyper and TNhaplotyper modules of the Sentieon package was used for calling variants in germline DNA and tumour DNA (as well as cfDNA), respectively [36]. The SNP & Variation Suite (GoldenHelix Inc., Bozeman, MT, USA) [34] was used for annotating called variants. Annotation was used for determining the effect of each variant on genes and their coding proteins. Annotation was performed using RefSeq and ClinVar to determine pathogenic germline variants [37,38].

Variants were classified as pathogenic if they were loss of function (LOF) variants or missense variants identified as “pathogenic” or “likely pathogenic” in the ClinVar database and literature based on ACMG classification. Germline variants were validated and included if they had greater than 20% variant allele frequency (VAF) and had a minimum of 20x coverage. Loss of heterozygosity (LOH) analysis involving SNV and indels was performed using tumour DNA. We looked for another pathogenic mutation in the tumour cells in addition to the germline mutation or the deletion of the entire wild-type gene that would be represented as LOH. VAFs for all pathogenic germline variants were compared with that in their somatic match to determine if there were any LOH. We considered LOH if the VAF was greater than 60%. The VAF cut-off point of 60% is based on the formula VAF = (%tumour cells)/(%tumour cells + (2 × (100 − %tumour cells))), which takes into consideration that the deletion of the wild-type allele in hereditary ovarian cancer is an early phenomenon, and therefore, present in most/all tumour cells. This represents 75% purity of the tumour cells.

For determining TSVs, all UTR and intronic variants were removed unless they were within five base pairs from a splice region. Somatic variants for each patient were classified as TSVs if they satisfied the following criteria: have a coverage of at least 100× in the tumour tissue sample with at least three reads supporting the variant allele and minimum VAF of 1% in tumour sample and a VAF less than 4% in the germline sample [39]. We implemented these criteria to focus on variants in the coding and splicing regions only and to verify true TSVs that were not an artifact or false call, given that TSVs can be present at low allele frequency in the tumour. Sequenced cfDNA extracted from post-surgery plasma samples was searched for TSVs as an indicator for the presence of ctDNA in the plasma. The presence of ctDNA was assigned if at least one TSV was identified in the sequenced cfDNA.

### 4.4. Statistical Analysis

We compared survival for patients according to residual disease status and ctDNA status. Patients were followed from the date of diagnosis until the date of first recurrence, death from another cause or date at last follow-up. Kaplan–Meier curves were used to generate crude survival curves, and the log-rank test was used to compare survival curves to calculate the *p*-value. A Cox proportional-hazards model was used to estimate the hazard ratios (HRs) and 95% confidence intervals (CIs) associated with variables of interest, including age at diagnosis (continuous), stage (II, III, IV), grade (I, II, III), and debulking status (residual/no residual disease). Survival analyses were conducted on the entire dataset, as well as on subgroups. Analyses were performed using SPSS version 26.0 (IBM SPSS Statistics, IBM Corp, Armonk, NY, USA).

## Figures and Tables

**Table 1:** Demographics of 48 patients with ovarian cancer.

Patient Characteristics	No. of Participants (%)
Age, mean (SD)	58.9 (10.1)
Menopausal Status	
Premenopause	7 (14.6%)
Perimenopause	3 (6.3%)
Postmenopause	37 (77.1%)
Unknown	1 (2.1%)
Histopathological classification	
Serous	47 (97.9%)
Mixed (serous/endometroid)	1 (2.1%)
Tumour stage	
Stage I	2 (4.2%)
Stage II	2 (4.2%)
Stage IIIA	2 (4.2%)
Stage IIIB	11 (22.9%)
Stage IIIC	28 (58.3%)
Unknown	3 (6.3%)
Tumour grade	
Low grade	1 (2.1%)
High grade	47 (97.9%)
Neoadjuvant treatment	
Received chemotherapy	0
Did not receive chemotherapy	45 (93.8%)
Unknown	3 (6.2%)
Adjuvant treatment	
Received chemotherapy	48 (100.0%)
Did not receive chemotherapy	0
Ascites	
Ascites present	30 (62.5%)
No ascites	15 (31.3%)
Unknown	3 (6.2%)
Ascites drainage	
Yes	26 (54.2%)
No	17 (35.4%)
Unknown	5 (10.4%)

**Table 2:** Identified pathogenic mutations in germline DNA.

Sample ID	Gene	Affected Transcript	Affected Protein	VAF in Germline (%)	Tumour Loss of Heterozygosity (LOH)	VAF in Tumour (%)
4	*BRCA1*	NM_007294.4: c.68_69delAG	p.Glu23fs	43.75	Yes	90.1
6	*RAD51C*	NM_058216.3: c.414G > C	p.Leu138Phe	47.9	Yes	77.9
	*CHEK2*	NM_007194.4: c.1100delC	p.Thur367fs	48.3	Yes	79.3
7	*BRCA1*	NM_007294.4: c.2709_2710delTG	p.Cys903_Glu904delinsTer	47.5	Yes	79
	*MSH6*	NM_001281492.1: c.3569_3572delCAAG	p.Ala1190fs	43.7	No	36.9
8	*BRCA1*	NM_007294.4: c.68_69delAG	p.Glu23fs	47.1	Yes	72.7
9	*RAD50*	NM_005732.4: c.2498_2499delAA	p.Gln833Argfs*11	50.2	No	46.8
14	*BRCA1*	NM_007294.3: c.5266C > T	p.Gln1756Ter	47.2	Yes	80.3
15	*BRCA2*	NM_000059.4: c.7806-2A > G	-	47.6	Yes	86.1
17	*BRCA1*	NM_007294.4: c.2241delC	p.Asp749fs	45.9	Yes	61.7
18	*BRCA1*	NM_007294.4: c.2241delC	p.Asp749fs	28.4	Yes	71.4
20	*BRCA2*	NM_000059.3: c.2409T > G	p.Tyr803Ter	50.3	Yes	82.5
23	*BRCA2*	NM_000059.3: c.1332_1333delTT	p.Ser445fs	45.6	Yes	71.1
27	*BRCA1*	NM_007294.3: c.5266C > T	p.Gln1756Ter	43.3	Yes	77.8
28	*BRCA1*	NM_007294.3: c.5266C > T	p.Gln1756Ter	47.7	Yes	71.8
29	*BRCA2*	NM_000059.3: c.8297del	p.Thr2766fs	42.4	Yes	75
33	*BRCA1*	NM_007294.4: c.4666C > T	p.Gln1556Ter	50.4	Yes	93.7
36	*BRCA1*	NM_007294.4: c.2241delC	p.Asp749fs	49.1	Yes	88.8
40	*BRCA2*	NM_000059.3: c.1332_1333delTT	p.Ser445fs	45.6	Yes	74.3
41	*BRCA1*	NM_007294.4: c.2999delA	p.Glu1000fs	48.9	Yes	82.2
44	*RAD51C*	NM_058216.3: c.397C > T	p.Gln133Ter	50.2	Yes	86.4
46	*BRCA1*	NM_007294.3: c.1439dupA	p.Asn480Lysfs	45	Yes	67.6
47	*BRCA2*	NM_000059.3: c.5946_5949delTGGA	p.Ser1982fs	46.3	Yes	75.2
48	*BRCA2*	NM_000059.3: c.1332_1333delTT	p.Ser445fs	47.4	Yes	75.6

**Table 3:** Cross-tabulation of the number of patients with germline and somatic pathogenic mutations.

	Germline Mutation				
**Somatic Mutation**		BRCA1	BRCA2	Other Genes	No Mutation
	BRCA1	0	1	0	1
	BRCA2	1	1	1	3
	P53	9	5	3	11
	Other Genes	1	0	0	4
	No Mutation	3	1	1	12

**Table 4:** Identified pathogenic tumour-specific variants (TSVs).

Sample ID	Gene	Affected Transcript	Affected Protein	VAF of TSV (%)
1	*TP53*	NM_000546.5:c.711G > T	p.Met237Ile	96.4
2	*TP53*	NM_000546.5:c.713G > C	p.Cys238Ser	80.3
3	*BRCA2*	NM_000059.4:c.8009C > T	p.Ser2670Leu	1.9
5	*TP53*	NM_000546.5:c.916C > T	p.Arg306Ter	43.5
	*BRCA1*	NM_007294.4:c.4357 + 1G > T	-	1.5
	*BRCA2*	NM_000059.3(BRCA2):c.3376G > T	p.Glu1126Ter	2.2
7	*TP53*	NM_000546.5:c.782 + 1G > A	-	65
8	*TP53*	NM_000546.6:c.524G > A	p.Arg175His	51.5
9	*BRCA2*	NM_000059.3:c.8297del	p.Thr2766fs	49.9
	*TP53*	NM_000546.6:c.524G > A	p.Arg175His	2.6
12	*TP53*	NM_000546.5:c.455del	p.Pro152fs	44.2
13	*TP53*	NM_000546.5:c.455del	p.Pro152fs	78.1
14	*BRCA2*	NM_000059.4:c.7007G > A	p.Arg2336His	1.3
	*TP53*	NM_000546.5:c.455del	p.Pro152fs	74.6
15	*TP53*	NM_000546.6:c.524G > A	p.Arg175His	87.8
16	*TP53*	NM_000546.5:c.455del	p.Pro152fs	44.3
17	*TP53*	NM_000546.6:c.844C > T	p.Arg282Trp	43.7
18	*TP53*	NM_000546.5:c.763A > T	p.Ile255Phe	67.2
20	*BRCA2*	NM_000059.4:c.5197_5198del	p.Ser1733fs	1.0
21	*TP53*	NM_000546.5:c.577C > G	p.His193Asp	54.6
23	*TP53*	NM_000546.5:c.537T > G	p.His179Gln	62
27	*MSH2*	NM_000251.2:c.1861C > T	p.Arg621Ter	1.2
	*TP53*	NM_000546.5:c.569C > T	p.Pro190Leu	73.1
28	*TP53*	NM_000546.5:c.711del	p.Met237fs	68.5
29	*TP53*	NM_000546.6:c.524G > A	p.Arg175His	65.8
	*BRCA1*	NM_007294.4:c.5096G > A	p.Arg1699Gln	1.2
31	*TP53*	NM_000546.5:c.469_471del	p.Val157delVal	64.2
32	*TP53*	NM_000546.6:c.524G > A	p.Arg175His	86.9
	*RB1*	NM_000321.3:c.1981C > T	p.Arg661Trp	88.8
33	*TP53*	NM_000546.6:c.1010G > C	p.Arg337Pro	1.7
34	*BRIP1*	NM_032043.2:c.1741C > T	p.Arg581Ter	1.0
35	*BRAF*	NM_004333.6:c.1796C > T	p.Thr599Ile	34.5
38	*BRCA2*	NM_000059.3:c.6814del	p.Arg2272fs	1.1
	*TP53*	NM_000546.5:c.844C > G	p.Arg282Gly	40.7
40	*TP53*	NM_000546.6:c.742C > T	p.Arg248Trp	74.6
41	*TP53*	NM_000546.5:c.488A > G	p.Tyr163Cys	84.3
43	*TP53*	NM_000546.5:c.713G > C	p.Cys238Ser	81.7
44	*TP53*	NM_001126112.2:c.216dup	p.Val73fs	54.9
47	*TP53*	NM_000546.5:c.725G > A	p.Cys242Tyr	80.5

## Data Availability

Genetic data will be available upon request.

## Supplementary Materials

The supporting information can be downloaded at: https://www.mdpi.com/article/10.3390/ijms241814388/s1.
