# Supplementary material for: Evaluating the Utility of ctDNA in Detecting Residual Cancer and Predicting Recurrence in Patients with Serous Ovarian Cancer
Source: Int J Mol Sci. 2023 Sep 21;24(18):14388. doi: 10.3390/ijms241814388 (PMC10532395; doi:10.3390/ijms241814388)
Supplement: Supplementary file 1 [file ijms-24-14388-s001.zip › ijms-2525609-supplementary.pdf]

Supplementary Materials

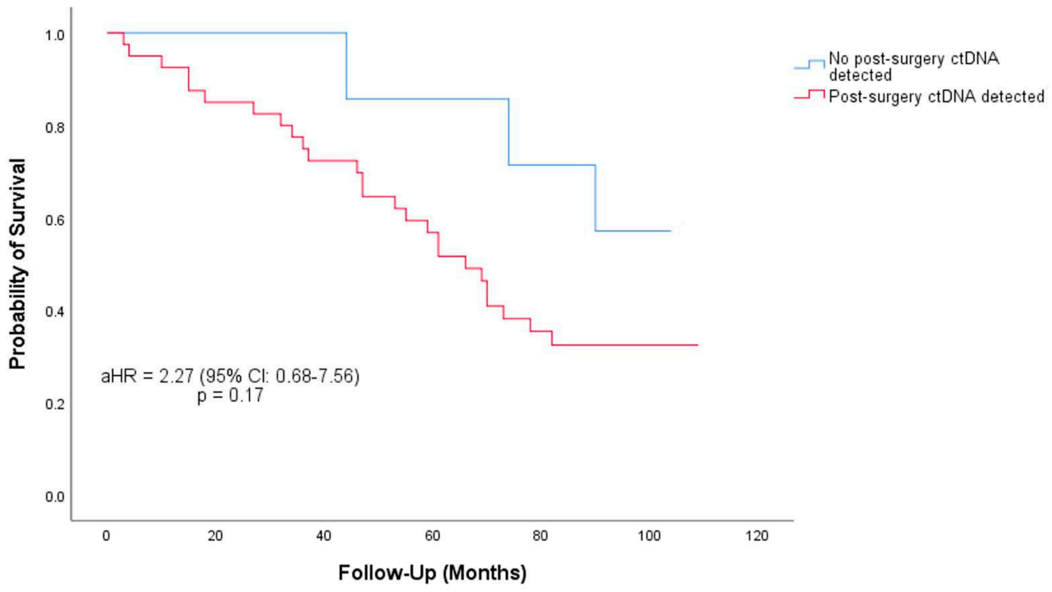

Figure S1. Kaplan–Meier survival curves for the overall survival of post-surgery samples with detectable (n = 40) and undetectable (n = 7) ctDNA. The log-rank test was used for comparing survival curves and calculating the p-value.

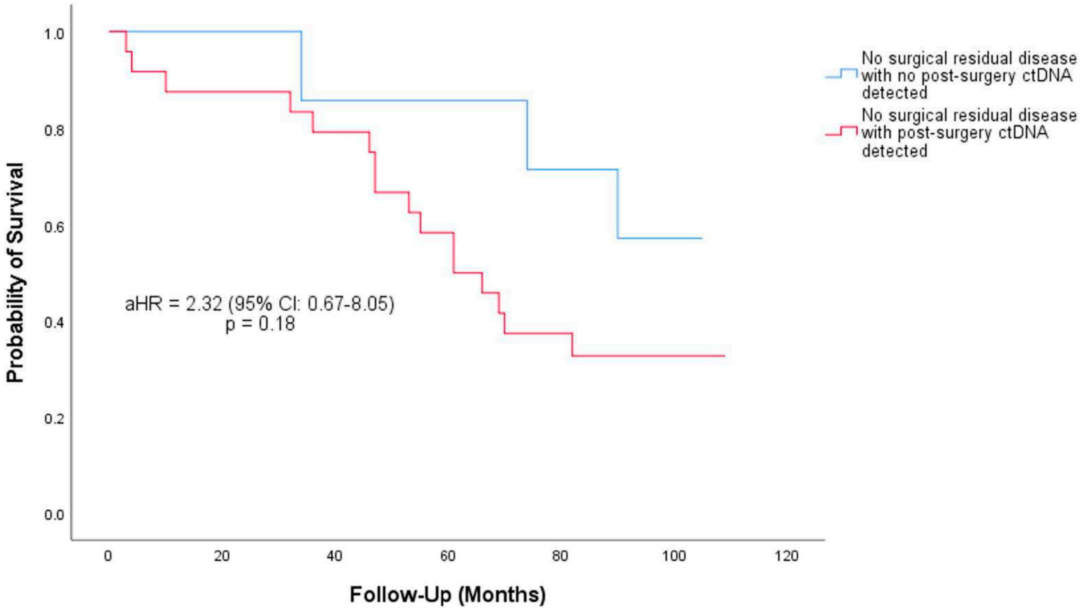

Figure S2. Kaplan–Meier survival subanalysis for the overall survival of patients classified as having no surgical residual disease with detected post-surgery ctDNA (n = 24) compared to those without detectable post-surgery ctDNA (n = 7). The log-rank test was used for comparing survival curves and calculating the p-value.

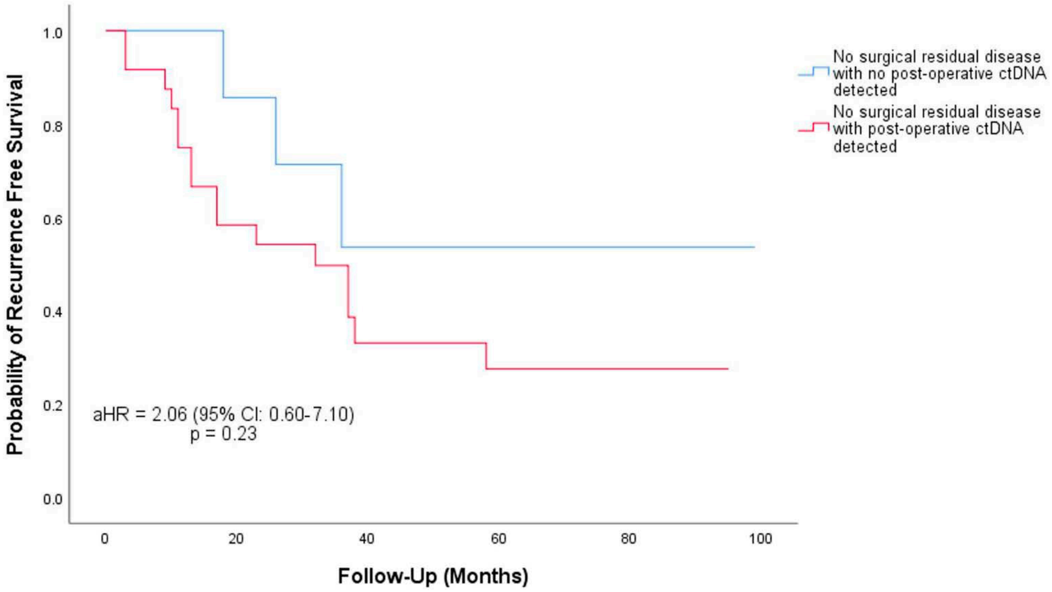

Figure S3. Kaplan–Meier survival subanalysis for the recurrence-free survival of patients classified as having no surgical residual disease with detected post-surgery ctDNA (n = 24) compared to those without detectable post-surgery ctDNA (n = 7). The log-rank test was used for comparing survival curves and calculating the p-value.
